# An investigation of household dogs as the source in a case of human bacteraemia caused by *Staphylococcus pseudintermedius*

**DOI:** 10.1080/20008686.2023.2229578

**Published:** 2023-07-03

**Authors:** Åse Östholm Balkhed, Robert Söderlund, Lotta Gunnarsson, Camilla Wikström, Helena Ljung, Carina Claesson, Stefan Börjesson

**Affiliations:** a Department of Biomedical and Clinical Sciences, Linköping University, Linköping, Sweden; b Department of Microbiology, National Veterinary Institute, Uppsala, Sweden; c Department of Animal Health and Antimicrobial Strategies, National Veterinary Institute, Uppsala, Sweden

**Keywords:** *Staphylococcus pseudintermedius*, bloodstream infection, bacteraemia, zoonotic infection, dog, canine

## Abstract

*Staphylococcus pseudintermedius* is a commensal and an opportunistic pathogen in dogs, and is also an opportunistic pathogen in humans. Here we report about a case of bacteraemia with a fatal outcome in a 77-year-old co-morbid male likely caused by a *S. pseudintermedius* and the investigation into the possible transmission from the two dogs in the patient’s household. The two dogs carried the same *S. pseudintermedius* strain, but this dog strain was unrelated to the strain from the patient. In contrast to the patient strain, the dog strain showed reduced susceptibility to several antibiotics and both dogs had received antibiotic treatment prior to sampling. So, it is conceivable that these treatments can have eliminated the patient’s strain between the transmission event and the dog sampling. It is also worth noting that the patient strain was positive for the *exp*A gene, which encodes an exfoliative toxin closely related to the *S. aureus* exfoliative toxin B. This toxin has been linked to canine pyoderma, but its effect on humans remains unknown. Transmission of *S. pseudintermedius* was confirmed in the household between the dogs. However, we could not verify that the dogs were the source for the *S. pseudintermedius* in the patient.

## Introduction

*Staphylococcus pseudintermedius* has been reported to be carried by up to 92% of healthy dogs and is considered part of the commensal microflora in dogs, but it is also a frequent opportunistic dog pathogen causing for example skin and soft tissue infections [1,2]. *S. pseudintermedius* has also been described as an emerging pathogen for humans with acquisition believed primarily to occur through zoonotic transmission, likely from dog contacts [3,4]. The first report of *S. pseudintermedius* as a cause of human infection was published in 2006, but it is more than likely that earlier published reports on *S. intermedius* in humans were in fact also *S. pseudintermedius* [5,6]. In humans *S. pseudintermedius* is primarily linked to skin, soft tissue, and sinonasal infections, but can also cause bacteraemia and there are also reports on infections related to implantations [4,7–14]. Occurrence of *S. pseudintermedius* has also been described in healthy humans, especially those in contact with dogs [15]. Since *S. pseudintermedius* is a coagulase-positive staphylococcus, sharing many phenotypic traits with *Staphylococcus aureus*, it is likely that it frequently has been, and is, misdiagnosed as *S. aureus* [7,16]

Methicillin-resistant *S. pseudintermedius* (MRSP) is also commonly found in veterinary settings, with human infections also reported [1,14,17]. Methicillin resistance in *S. pseudintermedius* is due to the *mec*A gene which also is the most frequent basis of methicillin-resistant *S. aureus* (MRSA). Initial findings of MRSP in Europe were associated with one clonal complex connected to the sequence type (ST) 71, but new clones and variants have since emerged [18,19].

*S. pseudintermedius* has previously been reported to cause bacteraemia on at least four occasions, but in most earlier reports sampling of companion animals in the household was not performed [3,8,9,12]. In this work, we came aware of a case of bacteraemia with a fatal outcome likely caused by *S. pseudintermedius*, and upon epidemiological investigation in the case it was revealed that there were two dogs living in the patient’s household, ages 12 years (Dog 1) and 10 years (Dog 2). The dogs were owned by his domestic partner and the partner informed us that both she and the patient had close contacts with both dogs. We therefore investigated if the dogs could be the source for *S. pseudintermedius* isolate from the patient and if the domestic partner also carried the same strain of *S. pseudintermedius.*

## Material and methods

### Presentation of the patient and the initial S. pseudintermedius isolation

The patient was a 77-year-old male with a history of cardiovascular disease, i.e. arterial hypertension, ischemic heart disease, atrial fibrillation, heart failure, venous and arterial insufficiency. Furthermore, he suffered from insulin-dependent type-2-diabetes with elevated HbA1c at 68 mml/mol, and chronic obstructive pulmonary disease. He presented to the emergency department with dyspnoea and pain located between the shoulders that had started the same morning. No fever was recorded. The patient had multiple venous leg ulcers. Chest X-rays demonstrated bilateral pleural effusion, most prominent on the right side, and drainage was applied. Blood tests from the initial presentation demonstrated inflammatory response with white cell blood count at 18.3 × 109/L, predominately neutrophils at 17.5 × 10^9^/L and c-reactive protein level at 267 mg/L. Samples for cultures were taken from blood, pleural effusion, urine, and nasopharynx. Treatment with cefotaxime twice daily was initiated. Echocardiography revealed no vegetation but signs of decreased systolic function and elevated pulmonary pressure. After four days with cefotaxime, culture results arrived and treatment was changed to cloxacillin three times daily intravenously for six days. As an outpatient, he was given oral flucloxacillin three times daily for eleven days. Nine days after finishing his antibiotic treatment the patient was re-admitted with dyspnoea, confusion, and aggravated heart failure. The patient died two days later. Unfortunately, on the last admission no cultures were obtained, nor were antibiotics administered.

In an aerobe blood culture bottle and an anaerobe bottle obtained from two puncture sites growth of gram-positive cocci was identified and using MALDI-TOF mass spectrometry these were shown to be *S. pseudintermedius*. Antibiotic susceptibility testing was performed by disc diffusion according to the protocol for *Staphylococcus spp*. and using NordicAST breakpoints (http://www.nordicast.org). The isolate was found to be susceptible to oxacillin, cefotaxime, clindamycin, tobramycin, erythromycin, fucidic acid, rifampicin, and linezolid. In cultures from pleural effusion *Staphylococcus pasteuri* and *Staphylococcus warneri* were detected. Approximately a year before the current investigation the patient also had a heel wound with growth of both *Streptococcus* group G and *S. pseudintermedius*, which unfortunately were not available for further characterisation at the time of this investigation.

### Sampling and cultivation of the dogs

The dogs were sampled by the local veterinarian five weeks after the patient’s death. From Dog 1 samples were collected from nose and lip fold, and from Dog 2 samples were collected from nose, throat, and ear, the sampling sites were chosen by the local veterinarian. Samples were sent to The National Veterinary Institute (SVA) and were on arrival cultivated on bovine blood agar, bromocresol purple lactose agar, and mannitol salt agar at 37°C for up to 48 h with suspected isolates analysed with a Bruker MALDI-TOF Biotyper System for species determination.

### Sampling and cultivation of the partner

A sample was also collected from the domestic partner by swab in anterior nares and cultivated on GC agar (EO-Labs Scotland) Hemoglobinagar (BD BBL, USA) Columbiaagar (Base CT Neogen, USA), Brilliance UTI agar (Oxoid, United Kingdom) and Chromagar Staph aureus (Chromagar, France).

### DNA extraction, genome sequencing and bioinformatics

DNA was extracted using EZ11 DNA Tissue Kit (QIAGEN, Hilden, Germany) according to the manufacturer’s protocol, and DNA concentrations were determined using QubitTM HS DNA Kit (Life Technologies, Carlsbad, CA, USA). All isolates were subjected to genome-sequencing using the Illumina Nextera XT kit and Illumina MiSeq paired-end 2 × 250 bp reads. Multi-locus sequence typing (MLST) was performed using the CGE online tool [20], and genome single nucleotide polymorphism (SNP) analysis was performed as previously described [21] with *S. pseudintermedius* ED99 (GenBank CP002478) as reference. Occurrence of *S. pseudintermedius* toxin genes, *lukI/S* (GenBank X79188), *siet* (AB099710), *SE-INT* (AB116378), *sec*_*Canine*_, (U91526), *exp*A (AB489850), *exp*B (AB569087) and SpEX [22] was checked by performing assembly by SPAdes v.3.11.1 followed by Pilon v.1.22 and BLASTn (https://blast.ncbi.nlm.nih.gov/). Genes encoding antibiotic resistance was determined with ARIBA v.2.13.3 [23] using a downloaded ResFinder database (https://cge.cbs.dtu.dk/services/ResFinder/, 2020-03-09).

### Antibiotic susceptibility testing

Minimum inhibitory concentrations (MIC) for 24 antibiotics were determined for the patient isolates and one isolate from each dog according to the standards for microdilution of Clinical and Laboratory standards institute [19] using Sensititre^TM^ EUST and STAFSTR panels (ThermoFisher Scientific).

### Ethical statement

The patient died before the study was performed, but the partner, and dog owner, has provided consent both orally and written. We have also fully anonymized the study and report.

## Results

### Identification of S. pseudintermedius and characterisation of the isolates

*S. pseudintermedius* could be identified from the two dogs from all samples collected, except from the nose of Dog 2. From the domestic partner no nasal carriage of *S. pseudintermedius* could be identified.

All isolates, *n* = 4, from the dogs belonged to the ST2023, while the single isolate from the patient belonged to ST21. The SNP-analysis confirmed that dog isolates and the patient isolate were unrelated to each other as the dog isolates clustered with pairwise distances of 1–20 SNPs, while the patient isolate was ≥8940 SNPs from dog isolates. All isolates carried *luk*S/F-I, *siet, Se-int* and *SpEX*, with the patient isolate also positive for *exp*A.

The patient isolate showed a MIC just above the suggested EUCAST Epidemiological cut-off (ECOFF) for Penicillin-G and the MIC results concurred with the previous disc diffusion test performed by the clinical laboratory. As the isolates from dogs showed high genetic relatedness one representative per dog was chosen for MIC-testing and both showed the same susceptibility pattern showing elevated MICs for Penicillin-G, fusidic acid, clindamycin, erythromycin, kanamycin, and streptomycin (Table 1). For patient isolate the only antibiotic resistance gene detected was *bla*Z, while the dog isolates also carried *ant*(6)-Ia, *aph*(3’)-III, *cfr*C, *erm*B and *fus*C in addition to *bla*Z.

**Table 1.** Minimum inhibitory concentration (MIC), mg/L, to tested antibiotics of S. pseudintermedius isolates from blood culture from the male patient and two dogs residing in his household. MIC determined using broth-microdilution and the SensititreTM EUST and STAFSTR panels. CLSI break-point for Staphylococci** (VEY01S) and EUCAST Epidemiological cut-offs (ECOFFs) (mic.Eucast.org).

		Chloramphenicol	Gentamicin	Kanamycin	Streptomycin	Rifampicin	Cefoxitin	Cephalothin	Oxacillin (NaCl)	Penicillin G	Fusidic Acid	Vancomycin	Clindamycin	Erythomycin	Mupirocin	Nitrofurantoin	Linezolid	Ciprofloxacin	Enrofloxacin	Tiamulin	Quinupristin/dalfopristin	Sulfamethoxazole	Trimethoprim	Trimethoprim/Sulfamethoxazole	Tetracycline
	**CLSI** **break-points**	Human ≥8	Human ≥16	**NA**	**NA**	**NA**	**NA**	≥8**	≥0.5**	**NA**	**NA**	Human ≥32	Dogs ≥4	**NA**	**NA**	Human ≥128	**NA**	**NA**	Dogs ≥4	**NA**	**NA**	**NA**	**NA**	Human ≥4/76	Human ≥16 dogs ≥1
	**EUCAST ECOFFs**	**NA**	>0.25	**NA**	**NA**	**NA**	**NA**	**NA**	**NA**	>0.03	**NA**	**NA**	>0.25	>0.5	**NA**	**NA**	**NA**	**NA**	>0.5	**NA**	**NA**	**NA**	**NA**	**NA**	>0.5
Strain ID	**Origin**																								
AB4107	Patient	8	≤1	≤4	≤4	≤0.016	0.5*	≤1*	≤0.25*	0.06*	≤0.5	≤1	≤0.12	≤0.25	≤0.5	≤16*	≤1	≤0.25	≤0.25*	≤0.5	≤0.5	256	≤2	0.5*	≤0.25*
AB4116	Dog 1	8	≤1	64	>32	≤0.016	≤0.25*	≤1*	≤0.25*	0.12*	4	≤1	>4	>8	≤0.5	≤16*	≤1	≤0.25	≤0.25*	≤0.5	≤0.5	256	≤2	0.5*	≤0.25*
AB4118	Dog 2	8	≤1	64	>32	≤0.016	≤0.25*	≤1*	≤0.25*	0.25*	4	≤1	>4	>8	≤0.5	≤16*	≤1	≤0.25	≤0.25*	≤0.5	≤0.5	256	≤2	0.5*	≤0.25*

*MIC determined by the STAFSTR panel, ** = *S. pseudintermedius* specific breakpoint.

## Discussion

In the current study, we could show that a *S. pseudintermedius* isolate from a patient was not directly related to the isolates recovered from the household dogs. Thus, transmission within the household from dogs to the patient could not be confirmed. A parallel outcome was described in a study by Nomoto et al. [12] which showed no relatedness between isolates from a patient and his dog. However, another study, by Blondeau et.al. [24], described that highly related isolates could be detected from a patient and a family dog. When reviewing available publications, it appears that transmission events between owners, veterinary staff and dogs do occur, but infrequently. For example, two studies from Spain and Hungary showed that the same strain was only shared by a single dog-owner pair out of 43 and 84 pairs sampled, respectively [25,26]. A Dutch study focusing on dogs infected with MRSP in households and veterinary clinics also concluded that transfer of MRSP occurs but that it was relatively uncommon [31]. It should be emphasised that the current case concerned a 77-year old co-morbid patient and elderly people with comorbidity factors and immunocompromised patients appear to be risk-groups for acquiring *S. pseudintermedius* infections [4,12,24–29].

We could however show that transfer likely had occurred between the two dogs in the household since they carried very closely related isolates. The veterinarian for the dogs also informed us that both dogs had been treated with antibiotics because of gingivostomatitis one week prior to being sampled, with Dog 1 given metronidazole and Dog 2 clindamycin. The use of metronidazole probably did not influence the occurrence of *S. pseudintermedius* as they are, like other aerobic and facultative anaerobic bacteria, intrinsically resistant to it. However, all isolates from the dogs showed increased MICs to clindamycin (Table 1) and carried the *erm*B gene. That only one strain was detected among the dogs could therefore potentially be connected to the antibiotic treatment the dogs received. The dogs were also unfortunately sampled five weeks after patient’s death, so it remains a possibility that one (or both) of the dogs originally carried the strain isolated from the patient but lost it because it was clindamycin susceptible (Table 1). In addition, it is interesting to note that the patient a year before the current investigation had a heel wound from which *S. pseudintermedius* could be cultivated. Thus making it possible that the patient might have acquired *S. pseudintermedius* from the dogs on several occasions, unfortunately the wound isolate were not available for further characterisation in this study.

Regarding the patient’s death it cannot be concluded that it was caused by a re-infection by a *S. pseudintermedius* since no cultures was taken upon re-admission. Upon re-admission to the emergency department with signs of organ dysfunction and a recent history of bacteraemia empiric antibiotic treatment should also have been initiated but was not done. Regarding the preceding antibiotic therapy with cefotaxime, cloxacillin and flucloxacillin it followed clinical practice for treatment of uncomplicated bacteraemia with methicillin-susceptible *S. aureus* at that setting. The *S. pseudintermedius* isolate from the patient also only showed reduced susceptibility to Penicillin-G, a phenotype that was confirmed genotypically with only the *bla*Z gene identified in the isolate. Clinical data on specific antibiotic therapy against *S. pseudintermedius* are however today lacking for human infections. In addition, *S. pseudintermedius* requires specific interpretation criteria for antibiotic susceptibility testing [30]. For example cefoxitin, which is commonly used to screen for MRSA, is not suitable for MRSP prediction; instead, oxacillin is recommended, and the defined oxacillin MIC, according to Clinical & Laboratory Standards Institute (CLSI) for MRSP is ≥0.5 mg/L, compared to ≥2 mg/L for MRSA.

All isolates in the current study carried the virulence-associated *lukS*/F-I, *siet*, *Se-int* and *SpEX* genes, which is not unexpected since these appear almost ubiquitous in *S. pseudintermedius* [7,26,27,32]. However, the patient ST21 isolate was also positive for the *exp*A gene, encoding an exfoliative toxin which is closely related to *S. aureus* Exfoliative toxin B, and has been linked to canine pyoderma [33]. A Spanish study also described an *S. pseudintermedius* ST21 with *exp*A from an apparent healthy human when investigating carriage and transfer of coagulase-positive Staphylococci in households with dogs, but that this ST21 was not present in the dog nor the other two humans in the household [25]. To our knowledge, the effect of this toxin on human cells has not been studied, but in a Swedish study focusing on dog bites 3 out of 13 identified *S. pseudintermedius* isolates were positive for *exp*A or the related *exp*B gene [7].

To summarize we could not determine that the dogs in a household were the source for *S. pseudintermedius* in a patient with a recent bacteraemia. The domestic partner of patient was also negative in the cultivation for *S. pseudintermedius*, but the lack of detection in the domestic partner might have been influenced by that no enrichment culture of the sample was performed. However, the two dogs did both carry *S. pseudintermedius* and these isolates showed high relatedness to each other, so transfer of *S. pseudintermedius* had occurred in the household at least between the dogs. Furthermore, the patient also had an earlier infection with *S. pseudintermedius*, which unfortunately was not available for typing. So it remains probable that the patient acquired *S. pseudintermedius* from dogs on several occasions either within or outside the household. One possibility is that the patients strain was lost in the household dogs due to the antibiotic treatment they received before the investigation was initiated. To better understand the zoonotic transmission dynamics of *S. pseudintermedius* further studies are needed, and one should also consider the potential existence of isolates that have an increased capacity to infect humans. In addition, specific antibiotic susceptibility cut-offs and therapy options for *S. pseudintermedius* should be considered as those used for *S. aureus*, or other staphylococci, may not be suitable. Based on the results from the current study, and those referenced, it might also be prudent to give advice on the risks of infection when having direct contacts with dogs to patients who are immunocompromised or have comorbidity factors.

## Data Availability

The data that support the findings of this study are available from the corresponding authors, [ÅÖB and SB], upon reasonable request. Sequence data is available from the European Nucleotide Archive under project accession number PRJEB56536, https://www.ebi.ac.uk/ena/browser/view/PRJEB56536.

## List of abbreviations

CLSI: Clinical & Laboratory Standards Institute
MLST: multi-locus sequence typing
MIC: Minimum inhibitory concentrations
MRSA: methicillin-resistant *S. aureus*
MRSP: Methicillin-resistant *S. pseudintermedius*
SNP: Single Nucleotide Polymorphism
ST: sequence type
